# Reconstruction of the Occipital and Parietal Congenital Defect with 3D Custom-Made Titanium Prosthesis: A Case Report with Four and a Half Years of Follow-Up and a Brief Review of Literature

**DOI:** 10.1155/2021/7027701

**Published:** 2021-10-20

**Authors:** Farnoush Mohammadi, Abbas Azari, Nariman Nikparto, Heliya Ziaei

**Affiliations:** ^1^Department of Oral and Maxillofacial Surgery, School of Dentistry, Tehran University of Medical Sciences, 1439955991, Kargar North St., Tehran, Iran; ^2^Department of Prosthodontics, School of Dentistry, Tehran University of Medical Sciences, 1439955991, Kargar North St., Tehran, Iran; ^3^School of Dentistry, Tehran University of Medical Sciences, 1439955991, Kargar North St., Tehran, Iran

## Abstract

Management of patients with congenital skull defects requires a multidisciplinary approach. Considering the defect's location and size, brain protection, and the cosmetic outcome makes such reconstructions challenging. Due to limited resemblance to skull contour and donor site morbidity of autogenous bone grafts, alloplastic materials are widely used for skull reconstructions. Titanium alloys have proper strength values, low infection rates, favorable osseointegration property, and excellent marginal adaptability when manufactured by computer-aided design (CAD) and computer-aided manufacturing (CAM). A 13-year-old female patient presented with congenital defects at the superior third of occipital bone and posterior thirds of the bilateral parietal bones. On CT scan, the exact size and shape of the defect were determined. Using CAD/CAM, a 3D virtual model of the prosthesis was designed and then printed with titanium alloy (TiAl6V4) via additive manufacturing method. The prosthesis was placed on the defect in a total surgery time of only 90 minutes. On 4.5 years of follow-up, the contour of the skull was ideal and the skin over the defect and neurologic status was intact. Due to their biocompatibility and rigidity, custom-made titanium prostheses are promising options for reconstructing complex skull defects.

## 1. Introduction

Skull defects are mainly due to congenital deformities, trauma, infection, or malignancy. Considering the location and size of the defect, along with brain protection and the cosmetic outcome, it makes such reconstructions challenging [1].

Autogenous bone grafts are assumed to be the gold standard for osseous reconstructions due to their immunocompatibility and low infection rates; however, donor site morbidity and lack of resemblance to skull contour are important drawbacks that limit their usage for the reconstruction of complex skull defects [2].

Due to these limitations, alloplastic materials that are biocompatible, inert, sterilizable, lightweight, noncarcinogenic, and cost-effective can be the option of choice for these reconstructions [3].

Whether prefabricated or custom-made, alloplastic materials are mainly made from titanium or polymathic methacrylate (PMMA) [3]. PMMA is economically affordable [3, 4]; however, it is not as strong as titanium [5]. Moreover, PMMA prostheses are more prone to infection, local irritation, foreign body reaction, and thermal damage during polymerization in the case being used as a cement for prosthesis adaptation. Furthermore, due to the native tissue ingrowth, prosthesis removal may be challenging [6].

Titanium alloys are amongst the most popular metals used in reconstructive procedures due to their high strength, biocompatibility, excellent contour, rigidity, low infection rate, favorable osseointegration property [7, 8], and excellent marginal adaptability, which can precisely fit into the complex geometry of skull defects, especially when 3D printed with CAD/CAM. These 3D-printed prostheses also have fewer radiographic artifacts in comparison to titanium meshes [9]. However, they are more expensive to design and manufacture [10].

Hence, the authors present a case of congenital skull defect with a history of several unsuccessful surgical treatments aimed at restoring the contour of skull. She was successfully treated with a 3D custom-made CAD/CAM titanium implant. For this reconstruction, the total surgery time was 90 minutes, which is 70% less than that of conventional cranioplasty procedures. Moreover, on 4.5 years of follow-up, excellent contour and the aesthetic outcome were evident.

## 2. Presentation of Case

The patient was a 13-year-old female with congenital skull defects at the superior third of occipital bone along with the posterior thirds of the bilateral parietal bones. Upon physical examination, the neurologic status was intact, and she did not have growth retardation. The genetic analysis showed no hereditary syndromes. She had her first reconstructive surgery in May 2008, at the age of 4 years, when the defect was bridged with a titanium mesh plate. Ever since that surgical treatment, she developed relentless petit mal seizures which were refractory to medications. The plate became exposed three months later, and she underwent several debridements in the following year, till the mesh was totally removed at the age of 5 leading to the resolution of seizures. She had not received any other medical treatment until the age of 13 when she was referred to our institution.

**Figure 1:**
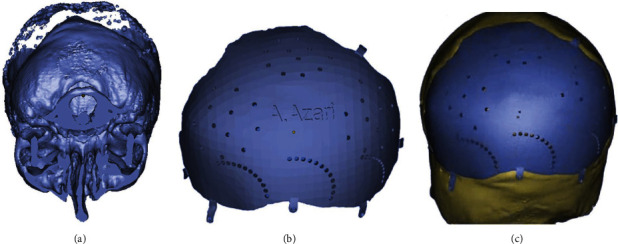
3D analysis. (a) CT scan shows the exact size and shape of the congenital defect. (b) The 3D virtual model of the prosthesis. (c) The 3D virtual model of the prosthesis adapted into the defect.

Because no further skull growth was expected [11], the patient was a candidate to receive the definitive prosthesis. A preoperative CT scan was obtained to determine the exact size and shape of the defect (Figure 1(a)). With CAD/CAM, a 3D virtual model of the prosthesis was designed and manufactured to match the borders of defect and provide ideal contour. The titanium alloy (TiAl6V4) was used to manufacture the prosthesis by implementing 3D printing via additive manufacturing method (Figures 1(b) and 1(c)).

**Figure 2:**
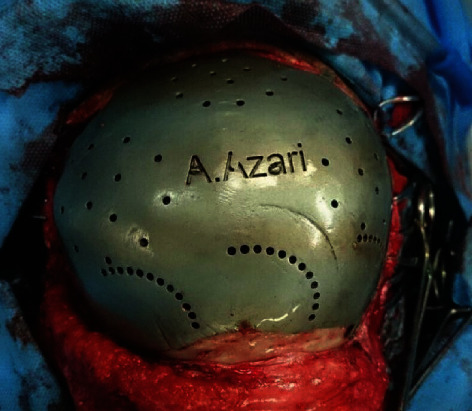
Intraoperative photograph showing the prosthesis fitness to the defect.

**Figure 3:**
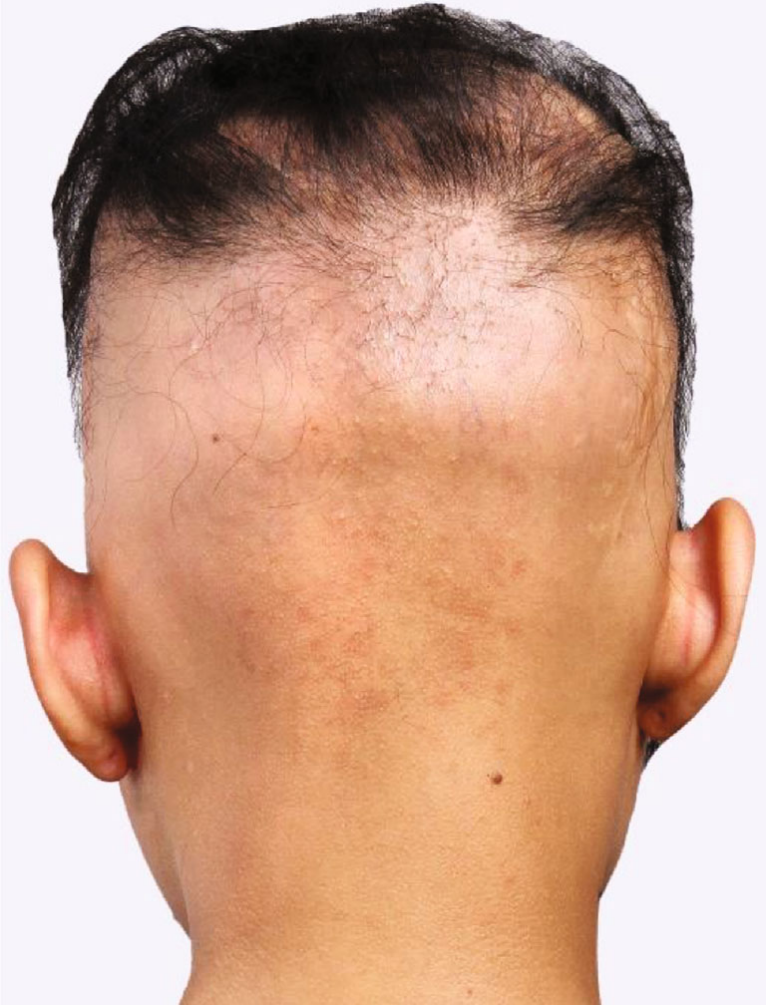
Four and a half years of follow-up. Note the perfect contour and healthy intact soft tissue at the age of 17.

For placing the prosthesis, bicoronal approach with subperiosteal dissection on the surrounding bony areas was performed. Extreme caution was exercised not to penetrate the dura during dissection between the galea and dura. The defect was totally exposed, and the dura remained intact. Then, the prosthesis was placed over the dura and fixed to the surrounding bone with titanium 2.0 mm miniplates. The ideal marginal adaptability and contour are shown in Figure 2. The incision was closed in three layers, taking care to approximate the tissues in a tension-free manner. The patient received intensive care for 24 hours and was discharged in the following week. On 4.5 years of follow-up (Figure 3), the skin over the defect remained intact with no signs of inflammation or neurologic deficits.

## 3. Discussion

Reconstructing complex skull defects is a challenge in craniomaxillofacial surgery. Major skull defects cannot be addressed with autografts since the contour of the skull is not similar to the common osseous donor sites such as the fibula, Iliac, or ribs [2]. Therefore, these defects may be best managed with a customized prosthesis. The 3D-printed PMMA or titanium prostheses were reported to have favorable outcomes. PMMA acrylic prostheses are more susceptible to infection [12] and tissue necrosis, which not only necessitates prostheses removal [13] but also jeopardizes the soft tissue over the defect, making further reconstructions more challenging [4]. Jaberi et al. reconstructed 70 patients with PMMA custom-made implants and reported a 24% complication rate [14].

To date, titanium is the most biocompatible material available in which the rigidity, anti-inflammatory, and antibacterial nature of this metal has made it the option of choice.

Conventional cranioplasty surgeries with prefabricated prostheses or titanium meshes usually take 4-5 hours due to the trial-and-error procedures to achieve a good fitness of the implant. By CADs, the complete procedure of the surgery is preplanned, and a customized prosthesis with excellent contour and fitness can be produced before the surgery. Therefore, not only there is no need for prosthesis adjustment during surgery but also the surgery time decreases considerably, and the outcome improves remarkably. By such a decrease in surgical time, the risk of postoperative complications such as severe pain, infection, and wound dehiscence decreases substantially. Our entire surgery lasted 1:30 hours which is approximately 70% less than conventional cranioplasty surgeries. Moreover, Saldarriaga reported the same, 85% reduction in surgery time with the similar method [15].

**Figure 4:**
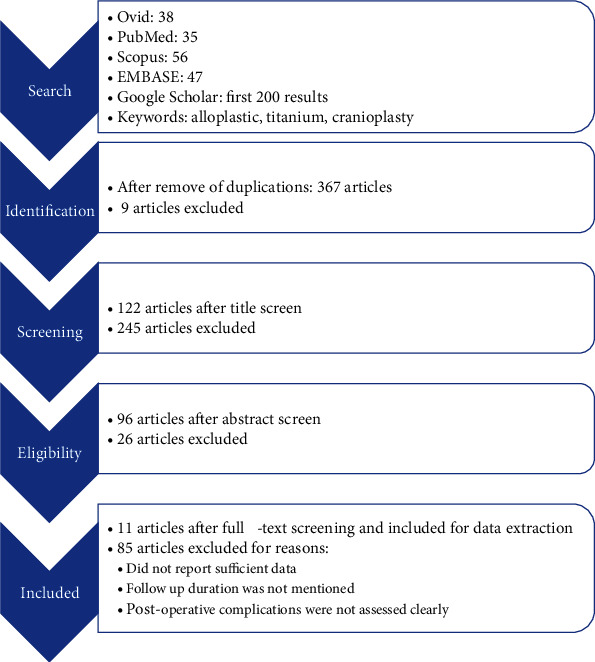
PRISMA flowchart. The details on selection process are reported.

To get a deeper understanding, a literature review was performed. Two authors (N.N. and H.Z.) independently conducted the electronic searches using Ovid MEDLINE/PubMed, Scopus, EMBASE, Google Scholar (first 200 results), and Cochrane Database, including articles published in March 2021. Alloplastic, titanium, and cranioplasty were used as keywords. The bibliographies of included studies were also searched for relevant articles. Preclinical studies, papers not describing cranioplasty techniques, and studies not reporting follow-up durations were excluded.  Figure 4 shows the PRISMA flowchart of selection criteria for this review. Finally, 11 studies with 214 reported cases were included.  Table 1 shows the extracted data, and Table 2 reports the analyses of data obtained from the included studies.

According to the results, the complication rate for titanium prosthesis reconstruction is relatively low. Among 215 cases with follow-up periods from 1 week to 8 years, 12 (5.6%) post-op complications and 2 (0.9%) prosthesis failures were reported. All studies reported excellent contour, acceptable rigidity, and high patient satisfaction rates. This rate of complications is by far more acceptable than that of PMMA prostheses.

The expenses of custom-made titanium implants are relatively high; however, the benefits definitely outweigh, such as decreased surgical time, shorter recovery and hospitalization, fewer postoperative complications, and less need to further corrective surgeries.

## 4. Conclusions

In conclusion, custom-made titanium implants can be considered the treatment of choice, which offers predictable aesthetic outcomes for the reconstruction of complex skull defects, with the low rate of complications and high aesthetics and patient satisfaction outcomes.

## Figures and Tables

**Table 1:** Summary of 11 included studies, reporting a total of 214 cases of skull reconstructions with custom-made CAD/CAM titanium prostheses.

Num.	Author/year	Patients	Gender	Age	Location	Titanium implant type	Follow-up	Post op complication
1	1998/E. Heissler [16]	15	12 male/3 female	21-35 years	Not mentioned	Solid	Mean 16.6 months	1 infection, 1 convulsion upon drainage removal
2	1999/J.JOFFE [17]	141	Not mentioned	6-67 years	Not mentioned	Solid	6 weeks to 1 year	1 failure
3	2009/Mario Cabraja [13]	26	Not mentioned	35.6	16 (frontotemporoparietal), 4(bifrontal), 3(frontal), 2(temporal), 1(frontoparietal)	Solid	Median 8.1 years	A transient palsy of the frontal ramus of the facial nerve postoperatively just in 1 case
4	2010/Jules Poukens [18]	1	Not mentioned	Not mentioned	Unilateral frontotemporoparietal	Solid	NM	No
5	2011/H. Sudhoff [9]	1	Male	48	Unilateral parietal, sphenoid, frontal, orbital rim, ethmoid, maxillary, zygomatic, nasal bones	Solid	1 week	No
6	2011/Juan Felipe Isaza Saldarriaga [15]	1	Male	13	Unilateral frontoparietal area	Solid	4 months	No
7	2014/S.A. Eolchiyan [19]	4	Female	24	Left fronto-orbital region	Solid	Mean 4.4 years	No
			Male	44	Right parietal-temporal region	Solid	Mean 4.4 years	No
			Female	23	Left fronto-orbital region	Solid	Mean 4.4 years	No
			Female	23	Both side frontal region	Solid	Mean 4.4 years	No
8	2015/Hyung Rok Cho [10]	3	Female	41	Left parieto-occipital area extended to right parietal area	Porous	6 months	No
			Female	32	Right frontotemporoparietal area	Porous	6 months	No
			Female	21	Left parietotemporal area	Porous	2 months	No
9	2018/Jae Yoon Kim [20]	1	Male	36	Unilateral forehead and glabella area	Solid	6 months	No
10	2019/Seong Hwan Kim [21]	2	Male	55	Right parietotemporal area	Solid	1 year	No
			Male	33	Right temporal area	Solid	NM	No
11	2019/Champeaux C [22]	19	12 male/7 female	47.4 ± 11.3	6: right side, 9: frontal side, 4: left side	Solid	Mean 1.2 year	31.6%: early postoperative complications, 1 lost to follow-up, 1 prosthesis removal, 63.1% no complication

**Table 2:** Summary of the demographic data and complication rates of the included patients.

N	214
Sex	
Female	7.47%
Male	14.02%
Not mentioned	78.50%
Age	38.93 ± 8.27
Follow-up	45.85 ± 40.01
Complication rate	5.61%
